# Vibration measurements of high-heat-load monochromators for DESY PETRA III extension

**DOI:** 10.1107/S1600577515005664

**Published:** 2015-05-09

**Authors:** Paw Kristiansen, Jan Horbach, Ralph Döhrmann, Joachim Heuer

**Affiliations:** a FMB Oxford Ltd, Unit 1 Ferry Mills, Oxford OX2 0ES, UK; b DESY, Deutsches Elektronen-Synchrotron Hamburg, Notkestrasse 85, 22607 Hamburg, Germany

**Keywords:** cryocooled DCM vibration

## Abstract

The requirement for vibrational stability of beamline optics continues to evolve rapidly to comply with the demands created by the improved brilliance of the third-generation low-emittance storage rings around the world. The challenge is to quantify the performance of the instrument before it is installed at the beamline. In this article, measurement techniques are presented that directly and accurately measure (i) the relative vibration between the two crystals of a double-crystal monochromator (DCM) and (ii) the absolute vibration of the second-crystal cage of a DCM. Excluding a synchrotron beam, the measurements are conducted under *in situ* conditions, connected to a liquid-nitrogen cryocooler. The investigated DCM utilizes a direct-drive (no gearing) goniometer for the Bragg rotation. The main causes of the DCM vibration are found to be the servoing of the direct-drive goniometer and the flexibility in the crystal cage motion stages. It is found that the investigated DCM can offer relative pitch vibration down to 48 nrad RMS (capacitive sensors, 0–5 kHz bandwidth) and absolute pitch vibration down to 82 nrad RMS (laser interferometer, 0–50 kHz bandwidth), with the Bragg axis brake engaged.

## Abstract 2

Vibration measurements of a cryocooled double-crystal monochromator are presented. The origins of the vibrations are identified. The minimum achieved vibration of the relative pitch between the two crystals is 48 nrad RMS and the minimum achieved absolute vibration of the second crystal is 82 nrad RMS.

## 1. Introduction   

The third-generation low-emittance synchrotron source PETRA III (Franz *et al.*, 2006 ▸) is currently in an upgrade phase. Eleven additional beamlines are being built. For beamlines P22, P23 and P64, high-heat-load double-crystal monochromators (DCMs), capable of preserving the stability of the storage ring, are needed. This requires the highest mechanical stability and sufficient cooling power to maintain the Si crystal lattice spacing even at 600 W heat load.

The P22, P23 and P64 beamlines will be equipped with a 2 m-long undulator with 32.8 mm period. P22 is suited for X-ray nano-spectroscopy in the energy range 3–50 keV. The purpose of P23 will be X-ray nano-diffraction (5–50 keV). P64 will be able to perform time-resolved- and bio-X-ray absorption spectroscopy at a flux of 5 × 10^12^ photons s^−1^ and within the energy range 4–45 keV. The monochromator described in this article will be solely used for EXAFS at P64; however, the DCMs of P22 and P23 are full duplicates.

The goal is to have vertical beam vibration at the detector that is smaller than the source size, approximately 10 µm RMS. For a 30 m distance between the DCM and detector this is equal to a relative pitch movement of the second crystal to the first crystal of roughly 150 nrad RMS. The large energy range (2.4–103.4 keV using Si[111] and Si[311]) and the demanded beam stability make very precise and rigid mechanical components necessary, whilst simultaneously requiring extended crystal movements. The cryocooling system is optimized to reduce vibration to the minimum.

The usual sources of DCM instabilities are: turbulent coolant flow, thermal in-equilibrities of the crystals and crystal cages, mount-induced vibration and, for directly driven DCMs, motor vibration (Chumakov *et al.*, 2004 ▸; Kelly *et al.*, 2013 ▸; Yamazaki *et al.*, 2013 ▸). In this article we investigate these issues by interferometric and capacitive methods adapted for this purpose.

## 2. Experimental setup   

The presented measurements were performed at the factory site of FMB Oxford in Oxford, UK.

### 2.1. Double-crystal monochromator   

**Figure 1:**
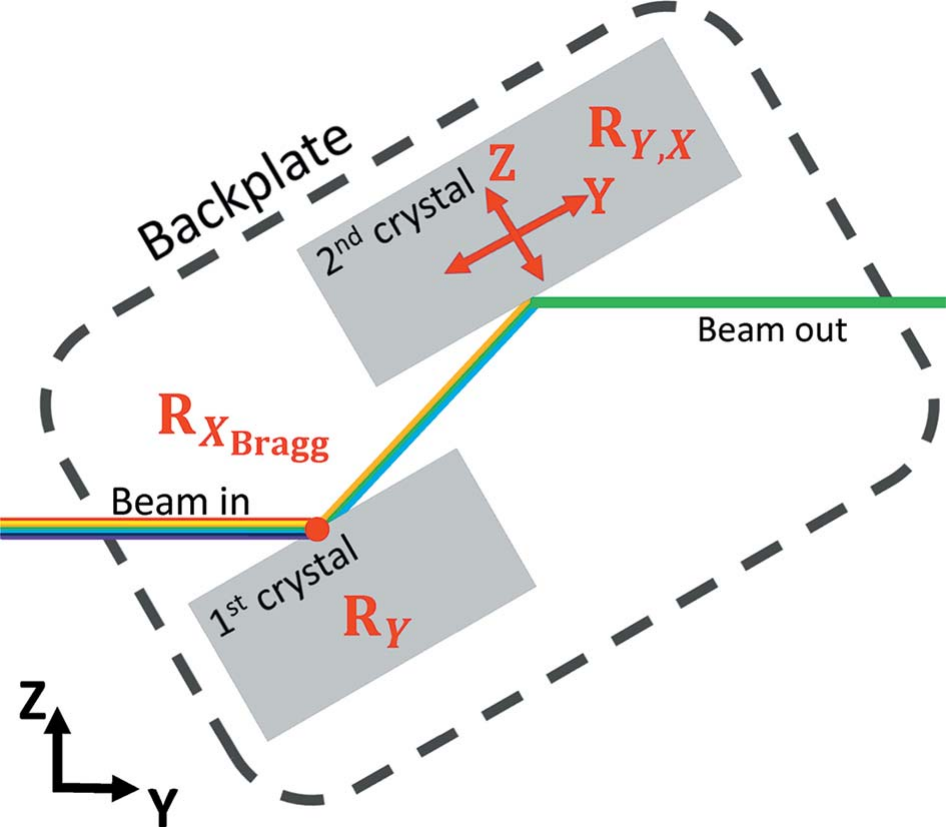
Schematic presentation of the DCM’s degrees of freedom (not to scale). The white beam passes the monochromator from left to right with a fixed offset of 21 mm.

**Figure 2:**
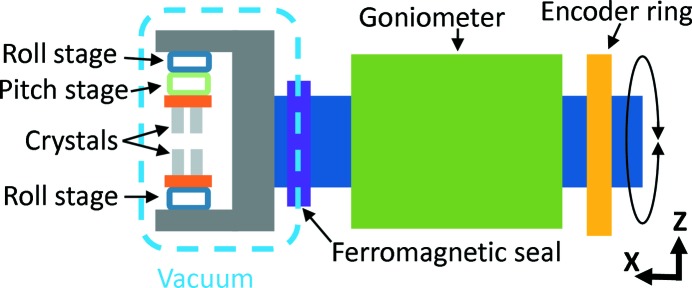
Schematic presentation of the Bragg drive shaft (not to scale), including flexures, roll for the first crystals and roll and pitch for the second crystals, and crystals and excluding all cooling lines.

The investigated monochromator is a highly versatile DCM (see Table 1 ▸ for motorized motions and ranges). Fixed-exit condition, 21 mm offset upwards, is provided for the Bragg range 2.1–56°. The DCM is fitted with two crystal pairs, Si[111] and Si[311] (see Figs. 2 and 4), which extend the monochromatic range to 2.4–103.4 keV. To change between the two crystal pairs the whole DCM is laterally translated. The first- and second-crystal cages are mounted on a common backplate that is rotated to vary the Bragg angle. Sealing of the rotational shaft is obtained by a ferromagnetic liquid seal. The rotational pivot point of the backplate is designed to coincide with the surface of the first crystal (see Fig. 1 ▸). In order to change the Bragg angle the backplate is bolted onto the rotational shaft of a direct-drive (no gearing) goniometer developed by FMB Oxford and RPI^1^.

**Figure 3:**
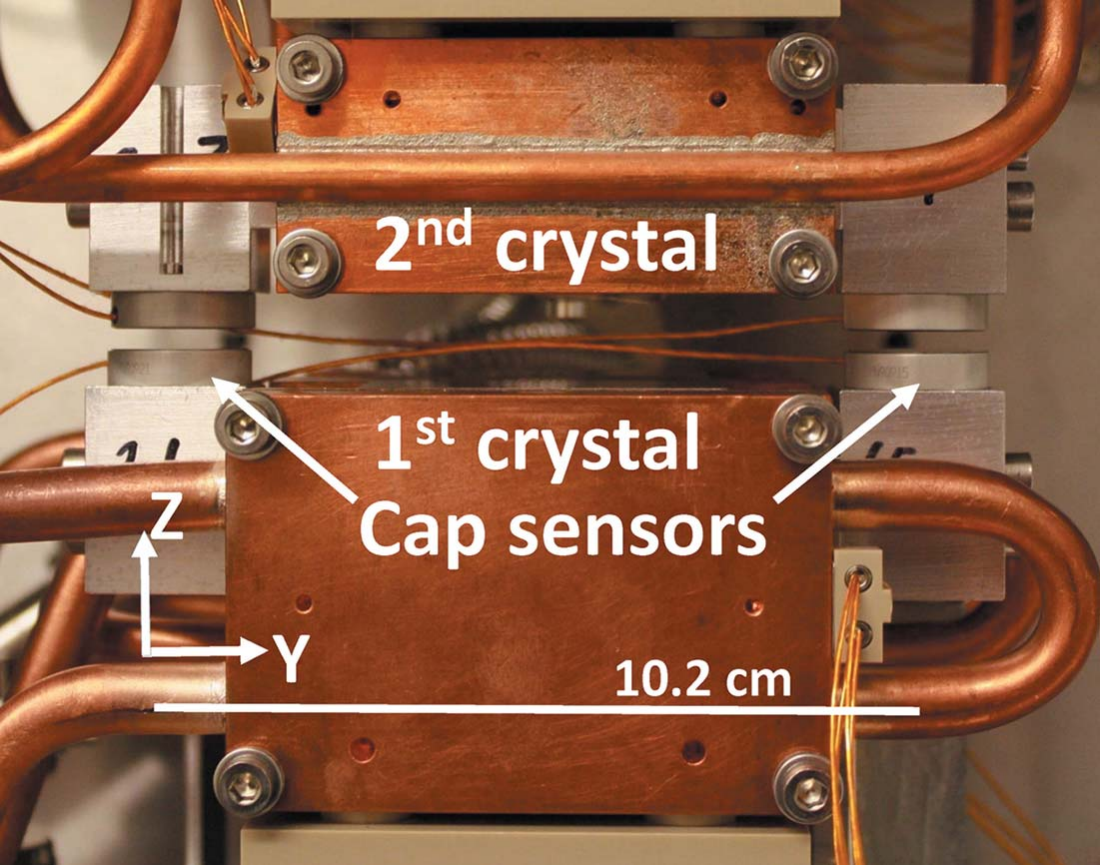
The mount of the capacitive distance sensors, here shown with a gap considerably larger than the nominal working gap of 100 µm.

**Figure 4:**
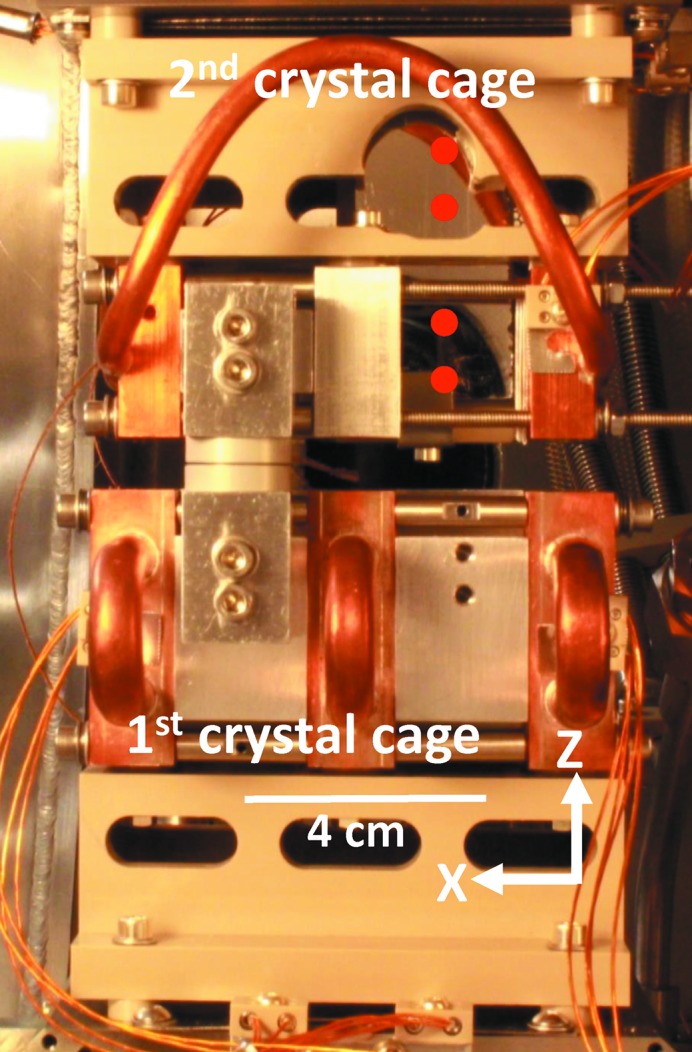
The mount of the mirror within the second-crystal cage. The four red dots illustrate interferometer beams. Note that the depicted PEEK block (Tavlet & Van der Burgt, 1994 ▸) supporting the second-crystal cage has been modified to accommodate the mirror.

Both the first and second crystal pairs are cryogenically cooled. This is realised by a FMB Oxford series D++ cryocooler^2^ pumping liquid nitrogen (LN) through Cu heat-exchanger blocks, with internal flow channels, that are clamped to either side of the crystals (see Figs. 3 and 4).

### 2.2. Bragg angle control   

The Bragg angle is controlled by a closed-loop system. The position is read from a high-resolution rotary encoder ring from Renishaw, that is clamped to the rotary shaft, with a Renishaw TONIC readhead (see Fig. 2 ▸). A 10.000x interpolater is used with the encoder ring and readhead to provide a commutation of 314.88 × 10^6^ counts per 360° revolution of the Bragg axis. Control of the motor is realised with a Delta Tau UMAC Turbo controller^3^ using pulse width modulation commutation.

The Bragg goniometer also includes a spring/pneumatic-actuated clamping brake. Pressurized air (6 bar) is needed to release the brake. The pressurized air used to release the brake is controlled by a solenoid valve operated by the control software. In case of power loss at the controller or a pressure drop in the air supply, the brake automatically engages.

### 2.3. DCM setup during measurements   

#### 2.3.1. Vacuum/cryocooling   

Prior to cooling of the dummy crystals the DCM pressure is brought down to ∼1 × 10^−5^ mbar by means of a turbo pump. At this pressure LN is led into the cooling circuit, actively cooling both the first and second crystals, and the system is left to thermally stabilize for ∼8 h. During the measurements the DCM is sealed off without any pumping. Once installed at the beamlines, vibration-free ion pumps will be solely used.

The cryolines connecting the cryocooler and the DCM were 9 m long. The LN pump worked at a frequency of 20 Hz with a target pressure of 1500 mbar above atmospheric pressure.

Within the DCM the LN flows to the first crystals through a coiled Cu pipe that is flexible enough to allow for the 1.2° roll. The cryogenic connection to the second crystals are flexible stainless steel tubes that are designed to reduce vibrations.

#### 2.3.2. Bragg motor   

The vibrational measurements are carried out either with (i) the motor servoing or (ii) the brake engaged and the motor off. The controller uses two cascaded control loops: one for the control of the three currents applied to the three-phase direct-drive motor, feed pulses at 8 kHz, and one for the position control using the encoder system, updating at 3.2 kHz.

### 2.4. Relative pitch vibrations   

In the ideal case where there is no relative pitch vibrations the beam will only be offset vertically while it maintains its direction when leaving the DCM. However, evidently there will be some relative pitch vibration, which will cause the beam to deviate from a parallel exit path. Thus relative pitch vibrations are of the highest concern, particularly for small beams on long beamlines.

In order to measure the relative pitch vibration between the two Bragg crystals two sets of Queensgate capacitive NanoSensors are used.^3^ ^4^ The capacitive sensors are mounted on a set of dummy crystals manufactured from Al (see Fig. 3 ▸). The capacitive distance sensors are capable of measuring with an accuracy of 0.1 nm with a bandwidth of 5 kHz.

### 2.5. Absolute pitch vibrations   

An absolute pitch vibration, where the angle between the two crystals is retained but the Bragg angle varies, has two effects on the monochromatic outgoing beam: (i) it will cause a change in the offset but the beam will retain a parallel path, and (ii) it will change the monochromated energy. However, as shown in §4.2, the effects of both are minute.

**Figure 5:**
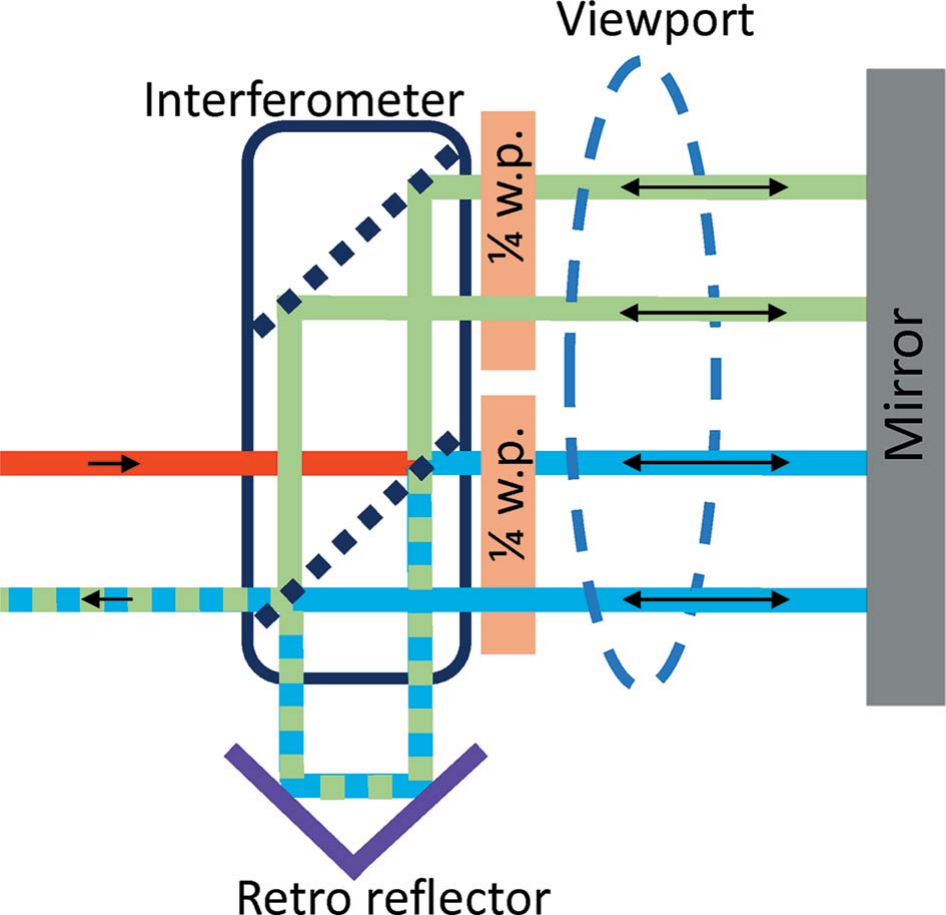
Schematic of the interferometer setup with quarter-wave plates allowing angular measurements of a mirror target (not to scale).

The measurements of the absolute vibration of the second-crystal cage are achieved by mounting a mirror in the crystal cage (see Fig. 4 ▸) that acts as the target of an interferometer outside the monochromator vessel (see Fig. 5 ▸). The interferometer setup incorporates quarter-wave plates mounted on the interferometer. By doing so a mirror can be used instead of a retroreflector as target (Duarte, 2003 ▸) (see Fig. 5 ▸). An XL-80 interferometer laser unit from Renishaw is used^5^, which can measure with an angular resolution of 5 nrad at a bandwidth of 50 kHz.

Optical viewports in the vessel are used at −2° and 30° Bragg. Since part of the laser beam is reflected by the viewport it is imperative that the laser beam does not cross the glass orthogonally, otherwise vibration of the viewport (*i.e.* the vacuum vessel) will be included in the measurement.

## 3. Results   

**Figure 6:**
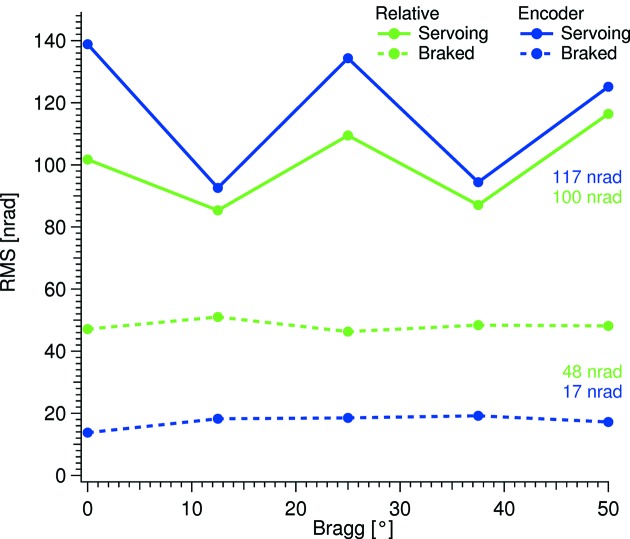
Relative pitch vibrations, in green, and absolute encoder vibrations, in blue. The colored numbers are averages.

The measured results are given in Tables 2 ▸ and 3 ▸ for the goniometer in a servoing and brake-engaged condition, respectively. The listed values are averaged over the measured motion ranges; full data are shown in Figs. 6 and 10. The frequency ranges describe the physical vibration that can be detected, hence the measurements were performed with twice the given maximum frequency. The measurements were carried out with the maximum bandwidth of the used instruments. The individual data points were collected over a period of 0.5 s. This time period was chosen to minimize air perturbations in the absolute measurements. The same measurement time applies to the relative measurements.

## 4. Discussion   

### 4.1. Relative   

**Figure 7:**
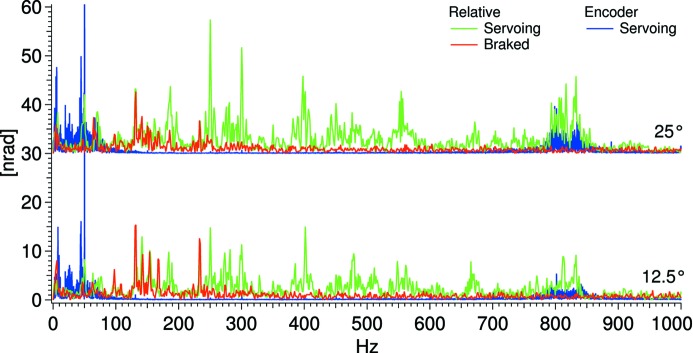
FFTs of the relative vibration (capacitive sensors) and the Bragg encoder vibration at 12.5° and 25°.

The relative pitch vibration between the two crystals, as the Bragg angle is changed, is displayed in Fig. 6 ▸. Under the assumption that a main cause of the relative pitch vibration is the vibration of the servoing Bragg motor, the vibration levels recorded with the incremental encoder are also displayed. The observed W-shapes of the servoing data are caused by different torques acting on the Bragg motor as the angle is varied. By the fast Fourier transforms (FFTs) shown in Fig. 7 ▸, a clear link between the servoing vibration around 830 Hz and the relative vibration at that frequency is seen, in particular at 25° Bragg. This link is less pronounced below 100 Hz, which indicates that the crystal cages are less susceptible to <100 Hz vibrations. The 50 Hz motor resonance can be reduced by altering the PID settings of the position control loop; this, however, will be at the cost of an overall increase of the vibrational level.

As seen, the relative pitch vibration is reduced by half when the Bragg motor servoing is stopped and the brake is engaged. It can be seen by the blue and red FFT traces in Fig. 11 that there are two sources, motor- and cooling-vibrations, as they show different spectra.

The most plausible cause of the 250–750 Hz features under servoing conditions in Fig. 7 ▸ are eigenstates of the backplate assembly.

**Figure 8:**
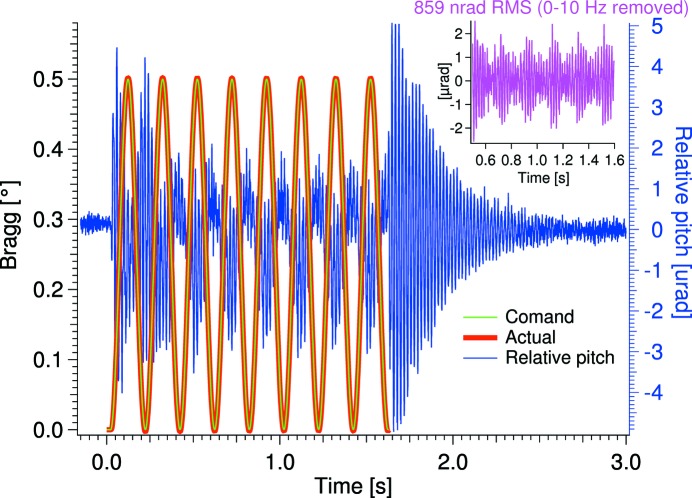
Relative pitch vibration during a 0.5° sinusoidal movement at 5 Hz. Insert: relative pitch vibration with externally caused vibration removed.

**Figure 9:**
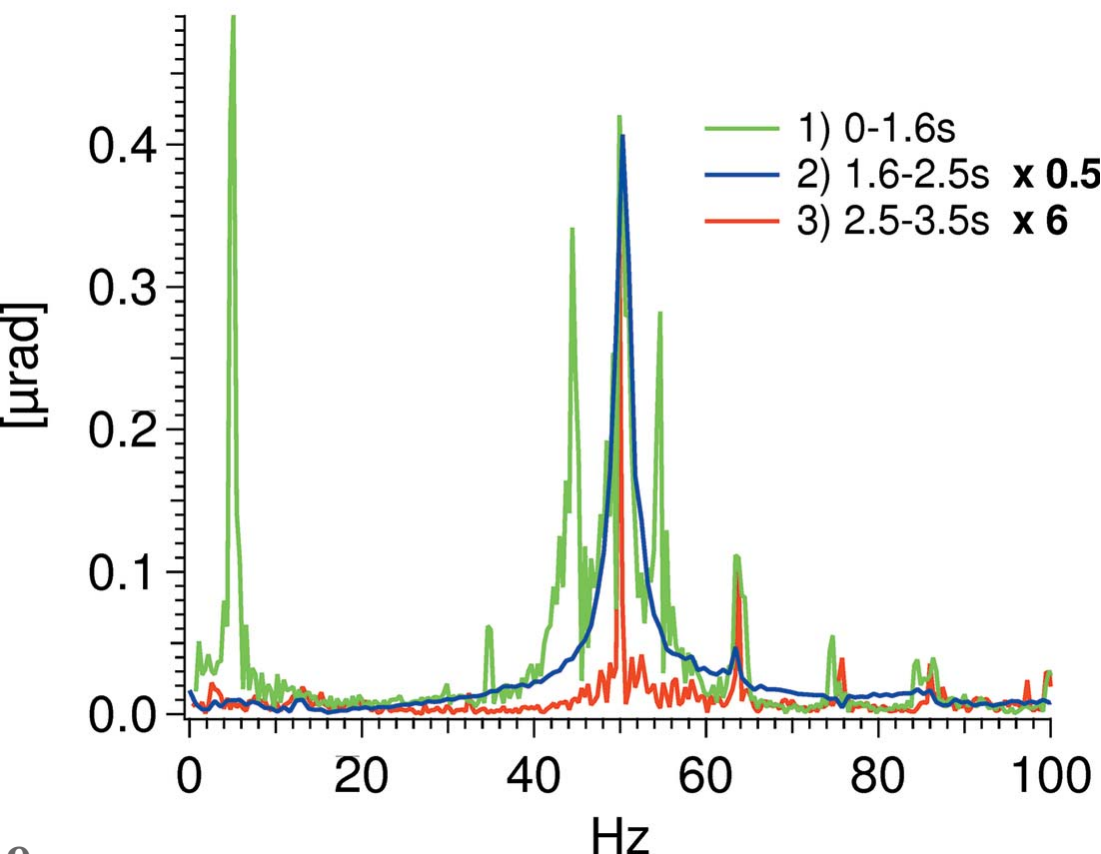
FFTs of the three stages of the dynamic test. Note the multiplication factors.

For testing the dynamic behavior of the crystal cages, measurements were performed while moving the Bragg drive. In Fig. 8 ▸ the relative pitch vibration during a 0.5° sinusoidal movement at 5 Hz is shown. The structure of the relative pitch vibration is seen to be dominated by a 50 Hz oscillation on top of a slow 5 Hz oscillation while the goniometer is being rocked (see Fig. 9 ▸). In Fig. 9 ▸, FFTs of the three stages of the dynamic test are shown: (1) during the rocking, green trace, (2) just after the rocking, blue trace, and (3) when stability is re-achieved, red trace. The large vibration seen just after the rocking is due to an abrupt transition between the rocking and standstill.

The 5 Hz vibration during the rocking is caused by the shaking of external cryolines. This assumption can be justified by the fact that the measured vibration under normal servo operation is reasonably low below 10 Hz (see Fig. 7 ▸). The external cryolines are hanging loosely in a setup not optimized for the rocking test. When these externally caused vibrations are removed (0–10 Hz) a dynamic stability at 5 Hz rocking of 859 nrad RMS, 10–2.5 kHz, is achieved (see the insert of Fig. 8 ▸). Since a typical Darwin width is about 5 µrad for Si 111 at 50 keV and 1 µrad for Si 311 at 103 keV, associated intensity variation can be neglected. Note that the two Darwin widths given here are the smallest attainable with the DCM.

### 4.2. Absolute   

**Figure 10:**
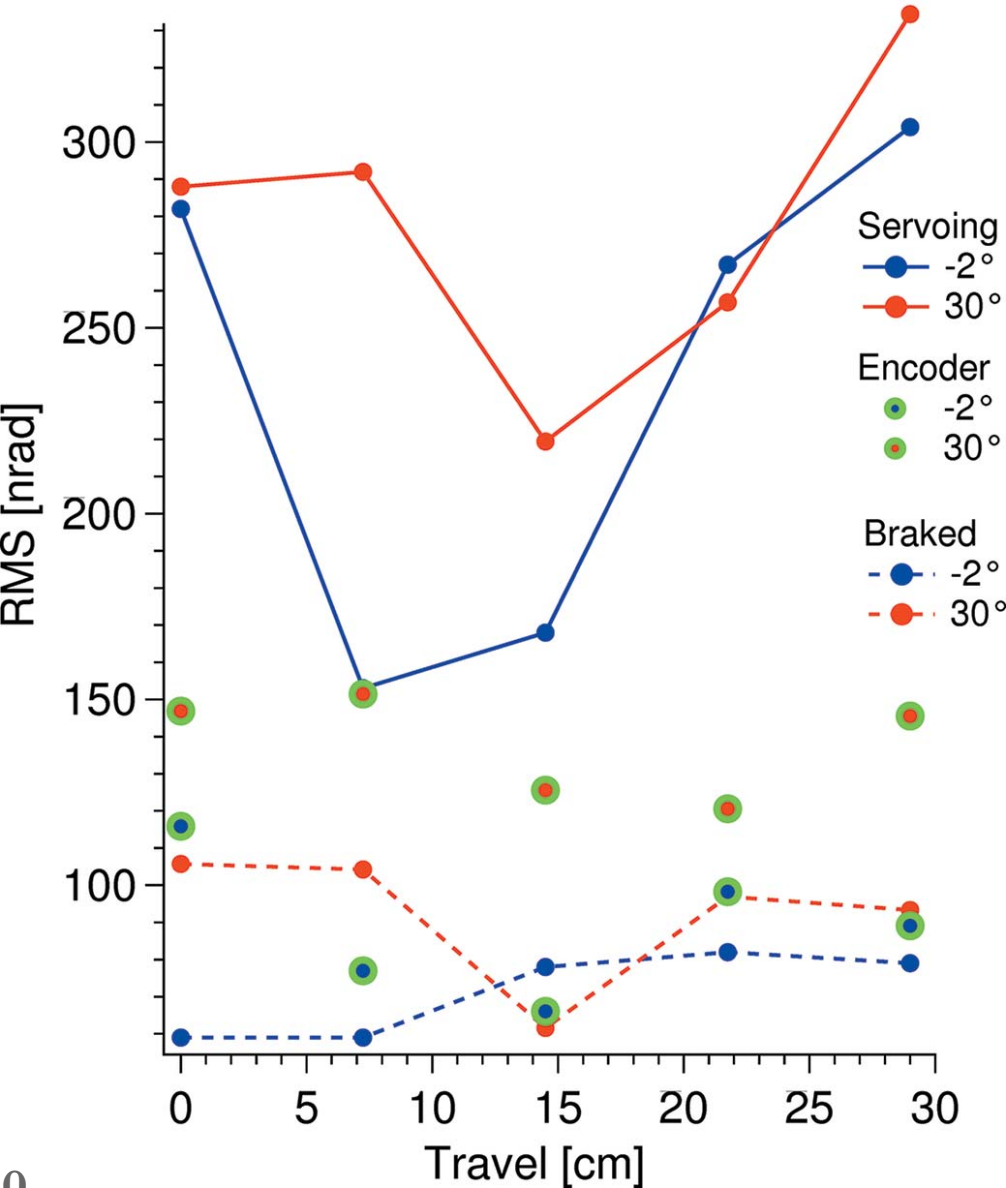
Absolute vibration of the monochromator measured at the second-crystal cage with the interferometer (red and blue) and the incremental Bragg encoder (green encircled dots). These encoder measurements were taken with the motor servoing. The encoder location is given in Fig. 2 ▸.

The RMS values of the absolute vibration of the second-crystal cage are displayed in Fig. 10 ▸ at five different positions as the crystal cage is moved in the parallel, *Y*, direction. During the measurements the DCM and the interferometer were mounted on a 3 m × 1.5 m mutual optical table that rested on air cushions. Although this setup gives a relatively good decoupling to the environment vibrations, some of the vibrational energy of the DCM will evidently migrate to the interferometer, which will raise the measured vibration level as the measurement is the sum of the interferometer and target vibrations.

The absolute vibration influences the energy stability and not the beam stability as the parallelity of the two crystals is not directly influenced hereby. For 300 nrad RMS absolute vibration measured at 30° the corresponding Si [111] Bragg reflex at 3.96 keV would jitter by 2 meV and change the offset by 2 nm RMS.

**Figure 11:**
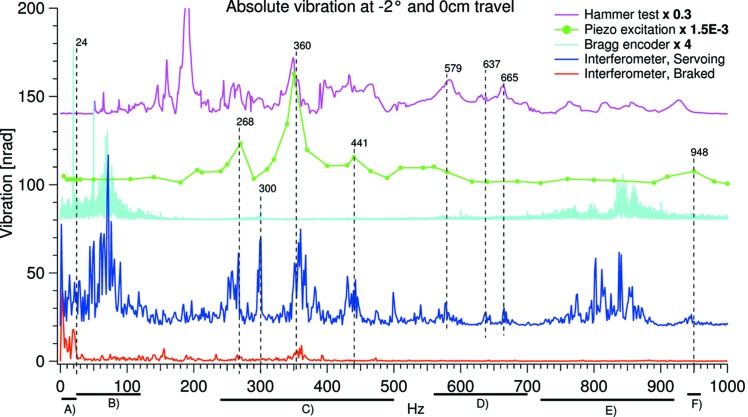
FFT analysis of the absolute vibration. Note the strongly varying multiplication factors of the three upper traces. The measurements in the marked frequency ranges (A) to (F) are connected to different vibrations sources; see text.

In order to quantitatively investigate the origin of the absolute vibration, the FFTs of the time domain data have been calculated and are shown in Fig. 11 ▸. The traces in the graph were measured at −2 or 0° Bragg and with the second-crystal cage at zero parallel travel, *Y* = 0 cm, where the capacitive sensors can be utilized. The red and blue traces show the absolute vibration measured with the interferometer: blue, with the motor servoing; red, brake engaged. The turquoise trace is measured by the incremental encoder with the motor servoing. Common for the three lower traces is that LN is flowing. The magenta trace is the resulting relative vibration from hitting the backplate with a hammer which supports both the first- and second-crystal cage (see Fig. 1 ▸).

The green trace in Fig. 11 ▸ shows the effect of an external periodical force on the second-crystal cage. The crystal cage is equipped with a piezo transducer, P-843 from Physik Instrumente^5^ ^6^, intended for fast and accurate correction of the relative crystal pitch. To find the pitch eigenmodes of the second-crystal cage we connected the piezo motor to an analog sine-wave source and monitored the resulting vibration amplitude with the capacitive sensors. This mainly yields information on the second-crystal cage as the first is not actively driven. However, some vibrational energy will evidently propagate through the backplate. The piezo was driven with a sine-wave of 0.5 V amplitude throughout the frequency range. The dots in the trace indicate the applied frequencies.

From Fig. 11 ▸ the origin of the absolute vibration with the motor servoing and LN flowing, represented by the blue trace, can be divided into groups as follows: (A) 2–24 Hz, flow/servoing induced as evident from the red and turquoise traces. (B) 24–120 Hz, servoing induced. (C) 240–500 Hz, eigenmodes of the second-crystal cage (excluding the 300 Hz peak that is clearly servo induced). There is no direct excitation source in this range, neither servoing- nor LN-frequencies, but the eigenmodes of the second-crystal cage are strong and could be excited from the higher servo frequencies. The main eigenfrequency of the second-crystal cage (360 Hz) is even present, by the red trace, when the motor is not servoing and the only excitation source is the flowing LN. (D) 560–700 Hz, eigenmodes of the whole backplate assembly, as seen by the magenta trace. There is no direct observable excitation sources in this region, but, due to the strength of the backplate assemblies eigenmodes, the needed excitation energy may come from higher frequencies. (E) 720–920 Hz, servoing induced vibrations. (F) ∼950 Hz, a combination of the second-crystal cage and servoing excitation.

The 190 Hz peak of the free vibration test (Fig. 11 ▸, magenta trace) does not coincidence with a peak in the blue trace. This could be due to the fact that the blue trace is measured with the interferometer, that exclusively detects pitch vibrations, whereas the free vibration test was measured with the capacitive sensors that will also detect yaw vibration.

### 4.3. Comparison of relative and absolute pitch vibration spectra   

**Figure 12:**
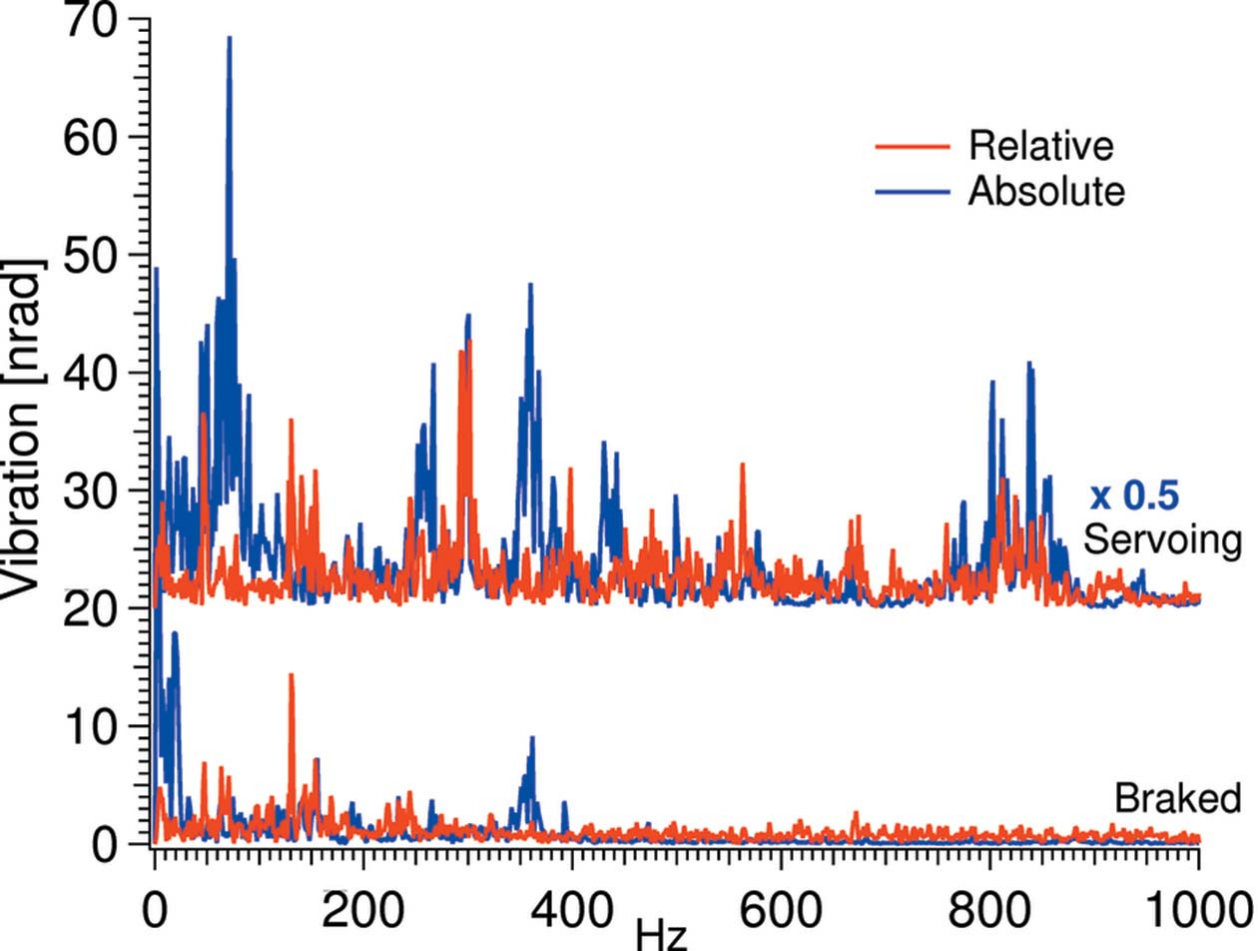
Relative *versus* absolute pitch vibration at 0° and −2° Bragg. Note that the servoing absolute trace has been divided by two.

Comparing the spectra of the relative and absolute vibration there are two striking differences (see Fig. 12 ▸): (i) the crystal cages are largely insensitive to relative pitch vibration up to 130 Hz; (ii) the crystal cages vibrate in-sync at the 360 Hz resonance of the absolute vibration.

## 5. Conclusion   

The monochromator shows excellent stability in the relative pitch vibration measurements, performed with flowing LN to both the first- and second-crystal sets. On average, 100 nrad RMS in the 0–2.5 kHz range is reached over the full Bragg range with the direct-drive goniometer servoing. When the brake of the goniometer is engaged, an average relative pitch vibrational level of 48 nrad RMS in the 0–2.5 kHz range is achieved.

The absolute pitch vibration is 250 nrad RMS (averaged over the parallel travel range of the second crystal cage) in the 0–25 kHz range with the direct-drive goniometer servoing and LN flowing. With the goniometer brake engaged an average absolute pitch vibrational level of 82 nrad RMS is achieved in the 0–25 kHz range. As pointed out before, the absolute vibration influences the energy stability and not the beam stability. For example, an energy jitter of 2 meV for the Si[111] Bragg reflex at 30° (3.96 keV) is expected.

In principle, the DCM could be used for fast scans, *e.g.* 0.5° Bragg at 5 Hz, with neglectable intensity variations of the monochromated beam as the relative vibration between the crystals stays well below the one-third Darwin width criterion for the full energy range of the DCM.

The main source of vibration is the servoing vibration of the direct-drive motor, when it is not in the brake-engaged condition, and the eigenmodes of the crystal cage excited by the flowing LN. Further developments will focus on the design of stiffer pitch and roll stages. This is expected to shift the resonance frequencies of these components upwards and to make them less susceptible to mechanical excitation.

## Figures and Tables

**Table 1:** Motion ranges of the investigated DCM

Motion	Range
Bragg rotation, *R*_*X*_Bragg__	63
First-crystal roll, *R*_*Y*_	1.2
Second-crystal roll, *R*_*Y*_	1.2
Second-crystal pitch, *R*_*X*_	1.2
Second-crystal parallel, *Y*	29cm
Second-crystal perpendicular, *Z*	2cm
DCM lateral, *X*	8cm

**Table 2:** Measured relative and absolute crystal vibration with the goniometer servoing The given relative pitch is an average over the full Bragg range.

Vibration	Frequency range (kHz)	RMS (nrad)
Relative pitch	02.5	100
Absolute pitch at 2	025	234
Absolute pitch at 30	025	278

**Table 3:** Measured relative and absolute crystal vibration with the goniometer brake engaged The given relative pitch is an average over the full Bragg range.

Vibration	Frequency range (kHz)	RMS (nrad)
Relative pitch	02.5	48
Absolute pitch at 2	025	71
Absolute pitch at 30	025	92

## Footnotes

1 RPI UK direct drive spindle assembly, http://www.rpiuk.com/.

2 FMB Oxford Cryocooler, http://www.fmb-oxford.com/product.php?product=45tab=1.

3 Tau Turbo UMAC, http://www.deltatau.com/DT_Products/ProductDetailDescription.aspx?CatID=800-TURBO%20UMAC.

4 Queensgate Nx nanosensor, http://www.nanopositioning.com/sites/nanopositioning.com/files/QUE1-NXNanoSensor.pdf.

5 Measurement optics for xl-80, http://www.renishaw.com/en/measurement-optics-for-xl-80--8427.

6 P 841 preloaded piezo actuator, http://www.physikinstrumente.com/product-detail-page/p-840-p-841-101000.html.
